# Supplementary material for: Unraveling bilayer interfacial features and their effects in polar polymer nanocomposites
Source: Nat Commun. 2023 Sep 15;14:5707. doi: 10.1038/s41467-023-41479-0 (PMC10504251; doi:10.1038/s41467-023-41479-0)
Supplement: Supplementary file 1 — Supplementary Information [file 41467_2023_41479_MOESM1_ESM.pdf]

Supplementary information for

Unraveling bilayer interfacial features and their effects in polar polymer nanocomposites

Xinhui Li[1], Shan He[2], Yanda Jiang[1], Jian Wang[1], Yi Yu[3], Xiaofei Liu[1], Feng Zhu[4], Yimei Xie[1], Youyong Li[3], Cheng Ma[4], Zhonghui Shen[1], Baowen Li[1], Yang Shen[2], Xin Zhang[1,6✉], Shujun Zhang[5✉] and Ce-Wen Nan[2✉]

[1]State Key Laboratory of Advanced Technology for Materials Synthesis and Processing, Center of Smart Materials and Devices, Wuhan University of Technology, Wuhan, Hubei, China
[2]School of Materials Science and Engineering, State Key Lab of New Ceramics and Fine Processing, Tsinghua University, Beijing, China
[3]Institute of Functional Nano and Soft Materials, Jiangsu Key Laboratory for Carbon-Based Functional Materials & Devices, Soochow University, Suzhou, China
[4]Division of Nanomaterials & Chemistry, Hefei National Research Center for Physical Sciences at the Microscale, CAS Key Laboratory of Materials for Energy Conversion, Department of Materials Science and Engineering, University of Science and Technology of China, Hefei, Anhui, China.
[5]Institute for Superconducting and Electronic Materials, Australian Institute of Innovative Materials, University of Wollongong, NSW, Australia
[6]International School of Materials Science and Engineering, Wuhan University of Technology, Wuhan, Hubei, China

✉E-mail: zhang-xin@whut.edu.cn (X.Z.); shujun@uow.edu.au (S.Z.); cwnan@tsinghua.edu.cn (C.-W.N.)

Supplementary Fig. 1 to 26

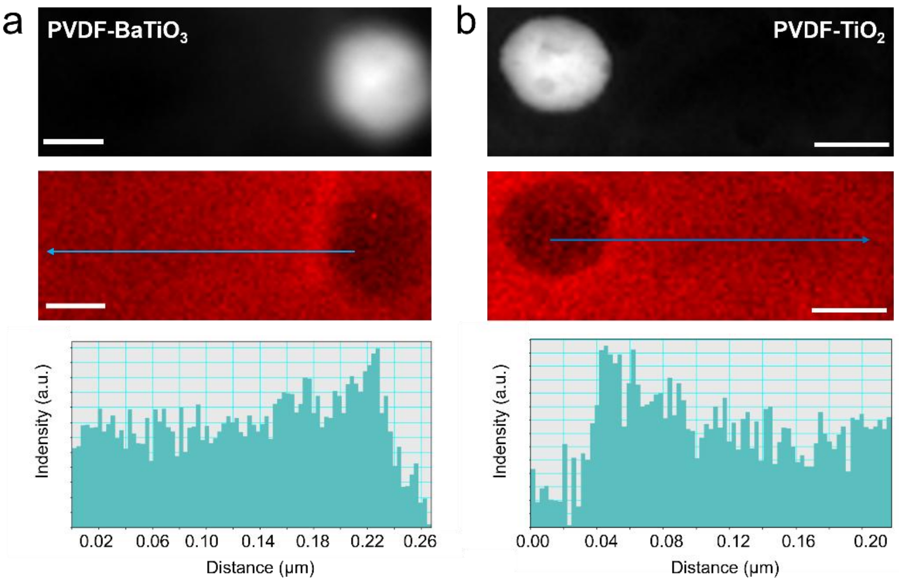

Supplementary Fig. 1 | The local density of interfacial polymers around the nanoparticle. a, The HADDF image (up) of a TiO_2 nanoparticle embedded in the PVDF matrix, and the corresponding element mapping (middle) and line profile (blue) of C signal (bottom) near the interface area. **b**, The HADDF image (up) of a $BaTiO_3$ nanoparticle embedded in the PVDF matrix, and the corresponding element mapping (middle) and line profile (blue) of C signal (bottom) near the interface area. The scale bar is 50 nm.

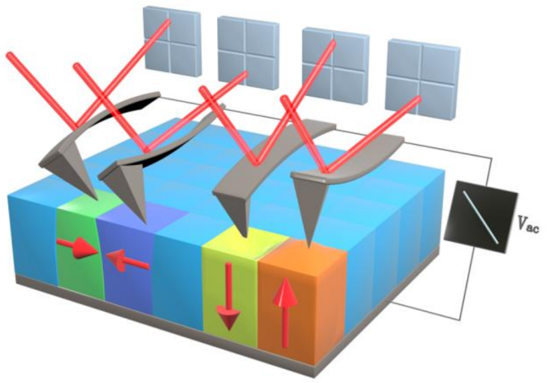

Supplementary Fig. 2 | The schematic illustration of the lateral and vertical PFM. The lateral and vertical PFM models allow the detection of dipole components from the in-plane and out-of-plane. In PFM test, the sample generally expands or contracts at the apex of the tip while an electrical voltage is applied to its surface. Such a deformation leads to mainly deflection, buckling or torsion of the cantilever. In vertical PFM and lateral PFM imaging, out-of-plane and in-plane polarization is measured by recording the tip-deflection signal and lateral motion of the cantilever due to bias-induced surface shearing, respectively.

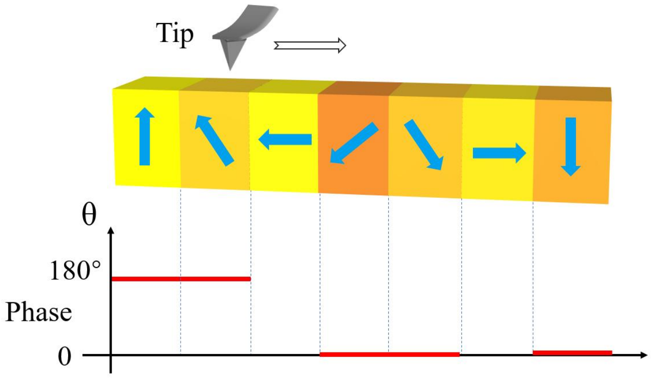

Supplementary Fig. 3 | The schematic illustration of phase signal in PFM. The phase of the measured response is proportional to the direction of the polarization, thus the sign of phase indicates the polarization orientation of the sample when an oscillating voltage is applied to the sample. The output of phase signal only exhibit two values, namely 0° and 180°, reflecting the vertical component of different dipole polarization. The phase of the dipoles with upward vertical component is 180°, while the phase of the dipoles with polarization pointing downward is 0°[1].

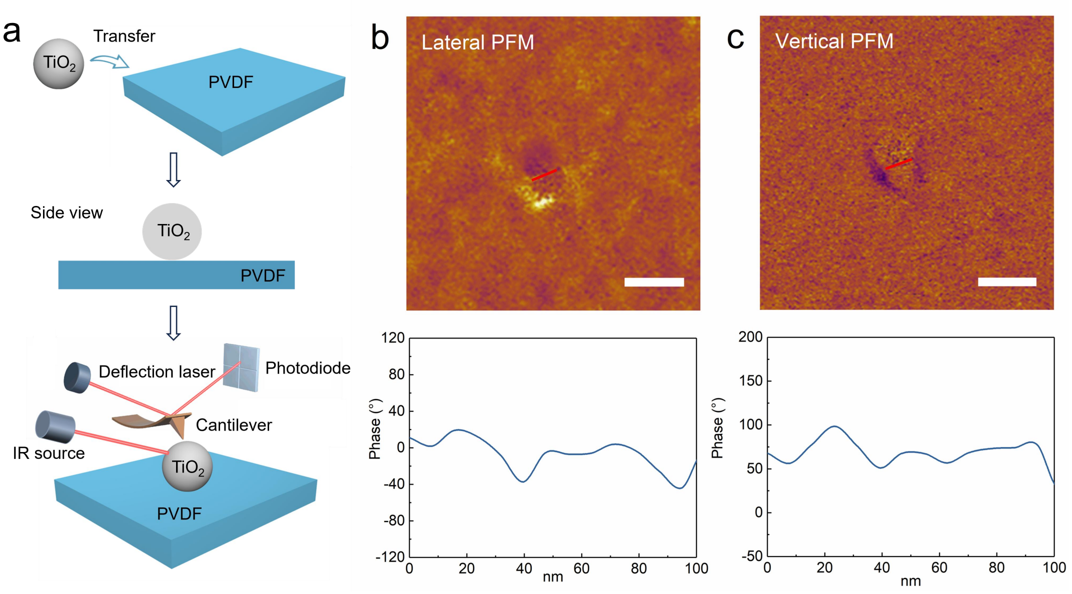

Supplementary Fig. 4 | The phase signal of the pristine TiO_2 nanoparticle. **a**, The schematic illustration of the pristine TiO_2 nanoparticle on the polymer surface. **b**, The Lateral PFM phase image (up) and corresponding line profile (bottom) of the pristine TiO_2 nanoparticle. **c**, The Vertical PFM phase image (up) and corresponding line profile (bottom) of the pristine TiO_2 nanoparticle. The scale bar is 200nm.

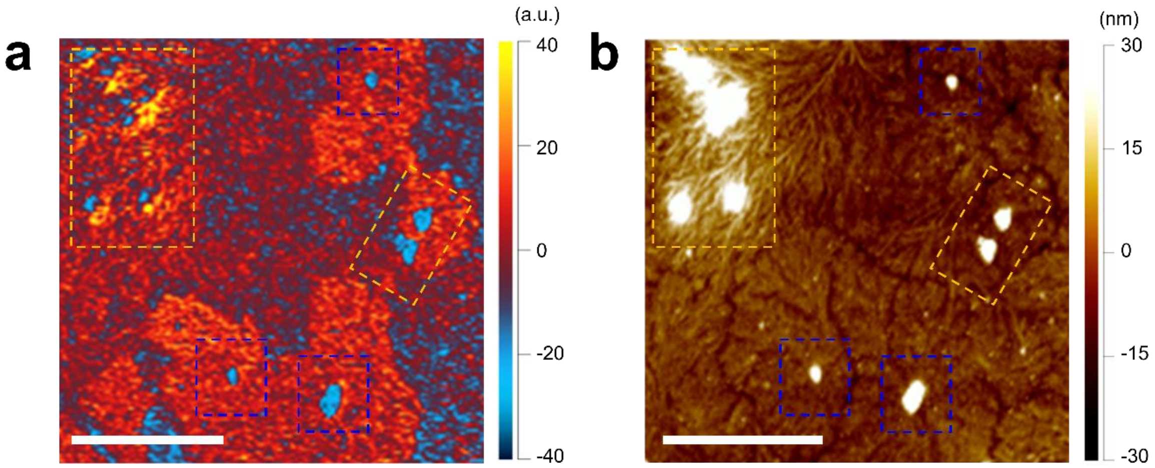

Supplementary Fig. 5 | Interfacial structure in PVDT-TiO$_2$ nanocomposite. a, **b**, The (a) AFM–IR chemical map with irradiation by a laser at 840 cm^{-1} and (b) corresponding AFM height image. The scale bar is 2 μm. The isolated interface is marked with blue rectangle and overlapped interface is marked with orange rectangle.

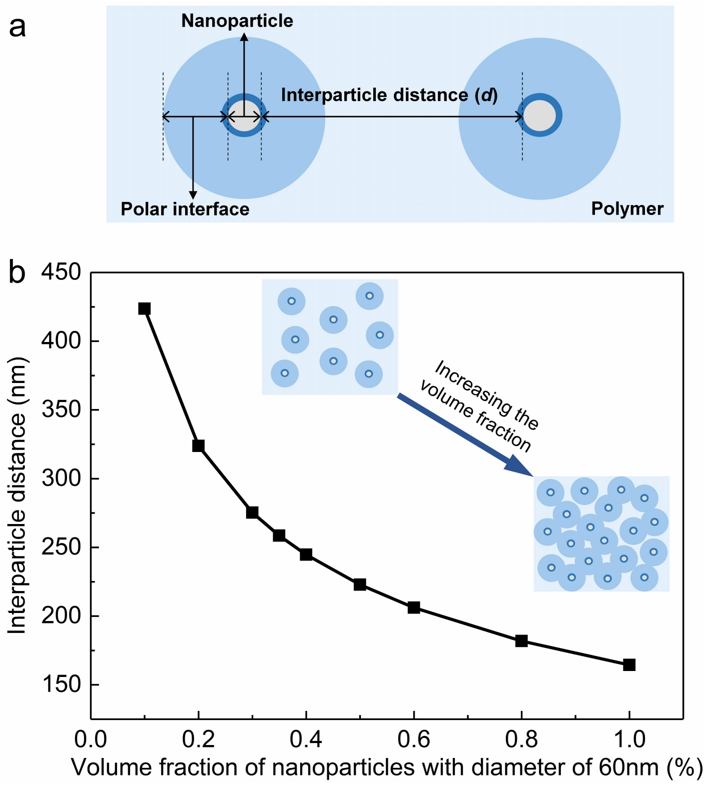

Supplementary Fig. 6 | The correlation between nanoparticle volume fraction and interparticle distance in polymer nanocomposite. a, The schematic illustration of the interparticle distance (d) between two neighboring nanoparticles **b**, The variation of interparticle distance with the volume fraction of nanoparticles (f_{particle}) in the polymer nanocomposite. By assuming a uniform nanoparticle dispersion, the relation between d and f_{particle} is expressed as: $f_{\text{particle}} = \frac{1}{6}\pi\left(\frac{D}{d+D}\right)^3$, and $d = D\left[\left(\frac{6f_{\text{particle}}}{\pi}\right)^{-\frac{1}{3}} - 1\right]$, where the D = 60 nm is the diameter of nanoparticles.[2]

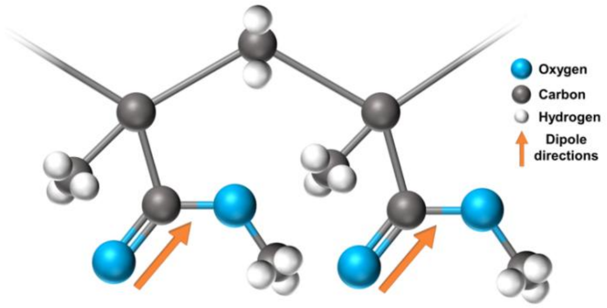

Supplementary Fig. 7 | Schematic illustration of PMMA polymer chain with mutual cis conformation of ester and methoxy groups, the dipoles are marked with orange arrows. The polarity of the PMMA chains greatly depends on the cis-trans isomerism of the ester and methoxy groups, the dipole moment of the ester group is located in the ester plane nearly antiparallel to the C=O bond. When two adjacent ester groups have the same orientations, the dipoles are parallel to each other and produce higher polarity of PMMA polymers[3].

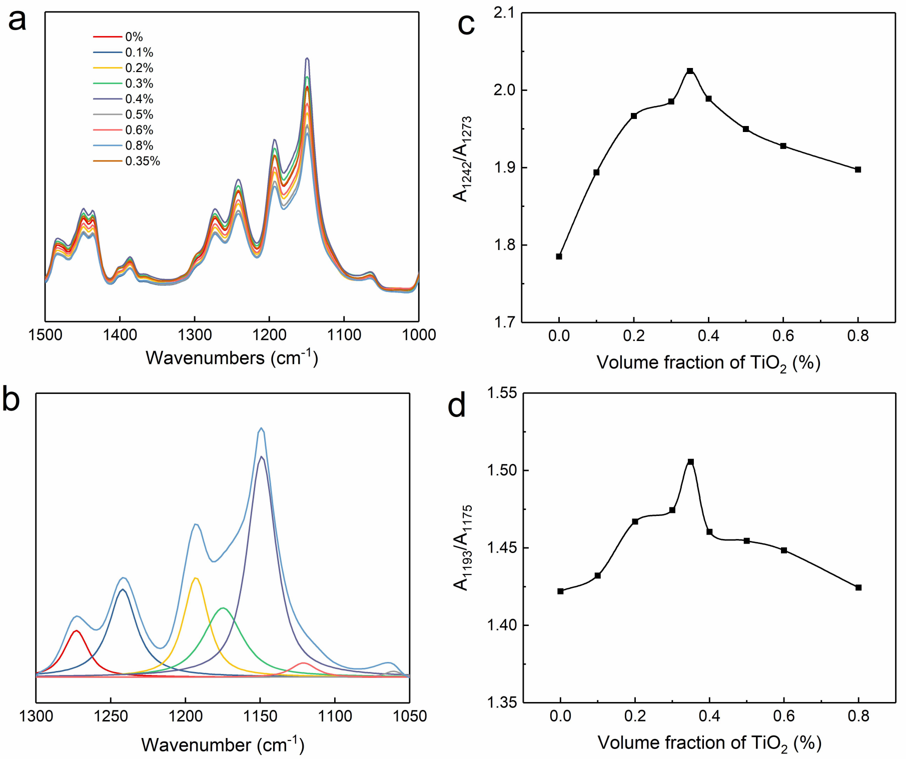

Supplementary Fig. 8 | The correlation between interfacial polar polymers and inter-particle distance in PMMA-TiO_2 nanocomposites. a, FTIR spectra of PMMA-TiO_2 nanocomposites with different volume fractions of nanoparticles. **b**, FTIR spectra in the region 1050-1300 cm^{-1} with fitted component bands at 1175 cm^{-1}, 1193 cm^{-1}, 1242 cm^{-1} and 1273 cm^{-1}. **c**, The calculated ratio of absorbance intensities between bands of 1242 cm^{-1} and 1273 cm^{-1} for nanocomposites with different volume fractions of nanoparticles. The cis and trans conformations of the ester group (C-C-O) are assigned to the 1242 cm^{-1} and 1273 cm^{-1} respectively[3,4]. **d**, The calculated ratio of absorbance intensities between bands of 1193 cm^{-1} and 1175 cm^{-1} in nanocomposites with different volume fractions of nanoparticles. The cis and trans conformations of the methoxy group (C-O-C) are assigned to the 1193 cm^{-1} and 1175 cm^{-1}, respectively[4,5]. The value of cis/trans conformation absorbance intensities for both ester (A_{1242}/A_{1273}) and methoxy (A_{1193}/A_{1175}) groups increases with increasing the volume fraction of TiO_2 nanoparticles, and reach the peak value at the volume fraction of 0.35%. The incorporation of TiO_2 nanoparticles leads to higher content of mutual cis conformation of ester and methoxy groups, which results in more parallelly orientated dipoles along the antiparallel direction of the C=O bond as well as higher polarities of PMMA polymers.

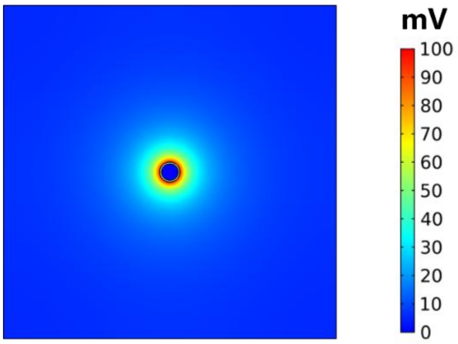

Supplementary Fig. 9 | The constructed surface electric potential near the nanoparticle. The distribution of electrical potential followed by Poisson and Boltzmann equations near a single TiO_2 nanoparticle. In the simulations, the electric potential following Poisson and Boltzmann equations was set onto the nanoparticle surface to build the charged surface, and the direction of the electric potential points perpendicularly to the particle surface.

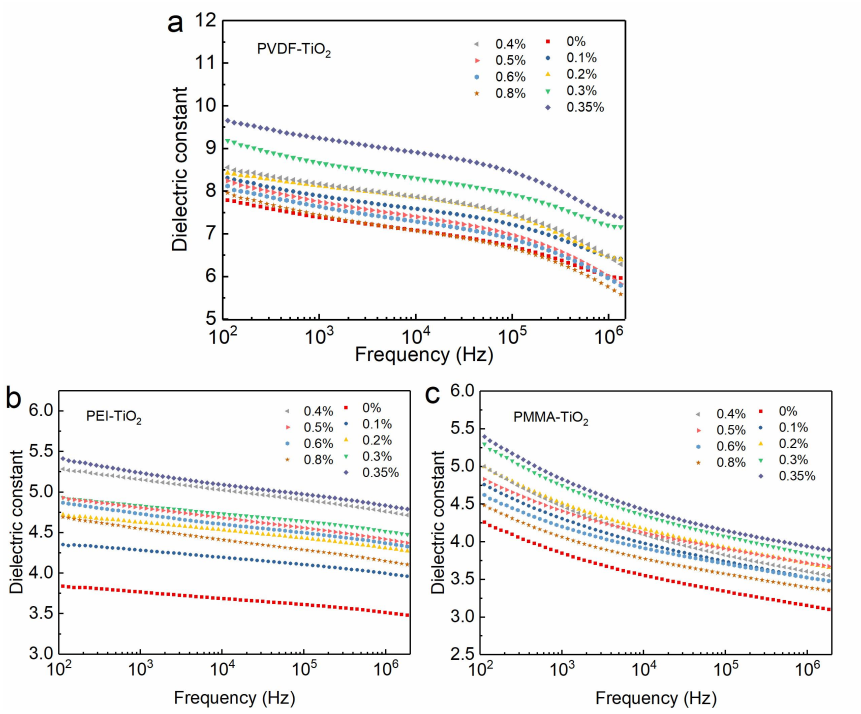

Supplementary Fig. 10 | The dielectric properties of polymer nanocomposites filled with varying volume fractions of nanoparticles. **a**, **b**, **c**, The frequency-dependent dielectric constant of (a) PVDF, (b) PEI, (c) PMMA based nanocomposites filled with varying volume fractions of TiO_2 nanoparticles.

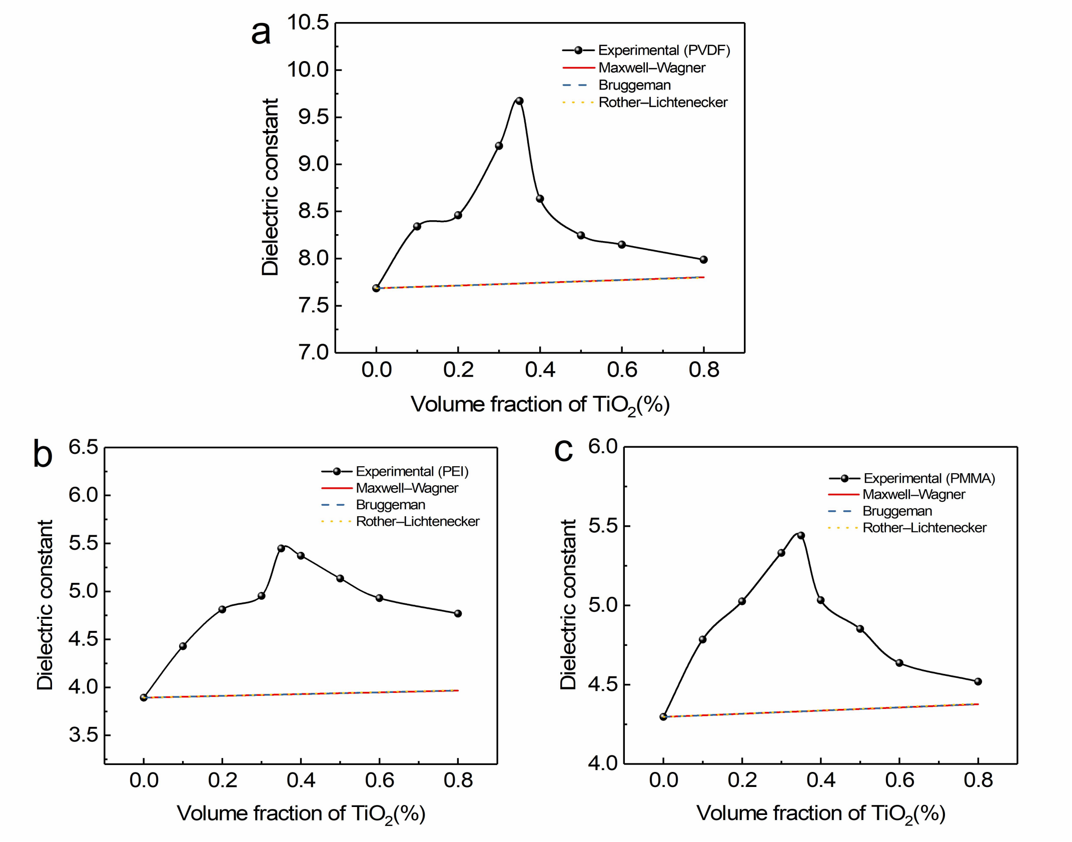

Supplementary Fig. 11 | The comparison of dielectric constant obtained by experimental data and classic two-phase mixture rules. a, b, c, The experimental and fitted dielectric constant of (a) PVDF, (b) PEI, (c) PMMA based nanocomposites filled with varying volume fractions of TiO_2 nanoparticles. The experimentally measured dielectric constants of nanocomposites show large deviations from the classic two-phase mixture rules including Maxwell-Wagner[6], Bruggeman[7], Rother-Lichtenecker[8] models, which exhibit unexpected enhancement at the dilute concentration of TiO_2 nanoparticles.

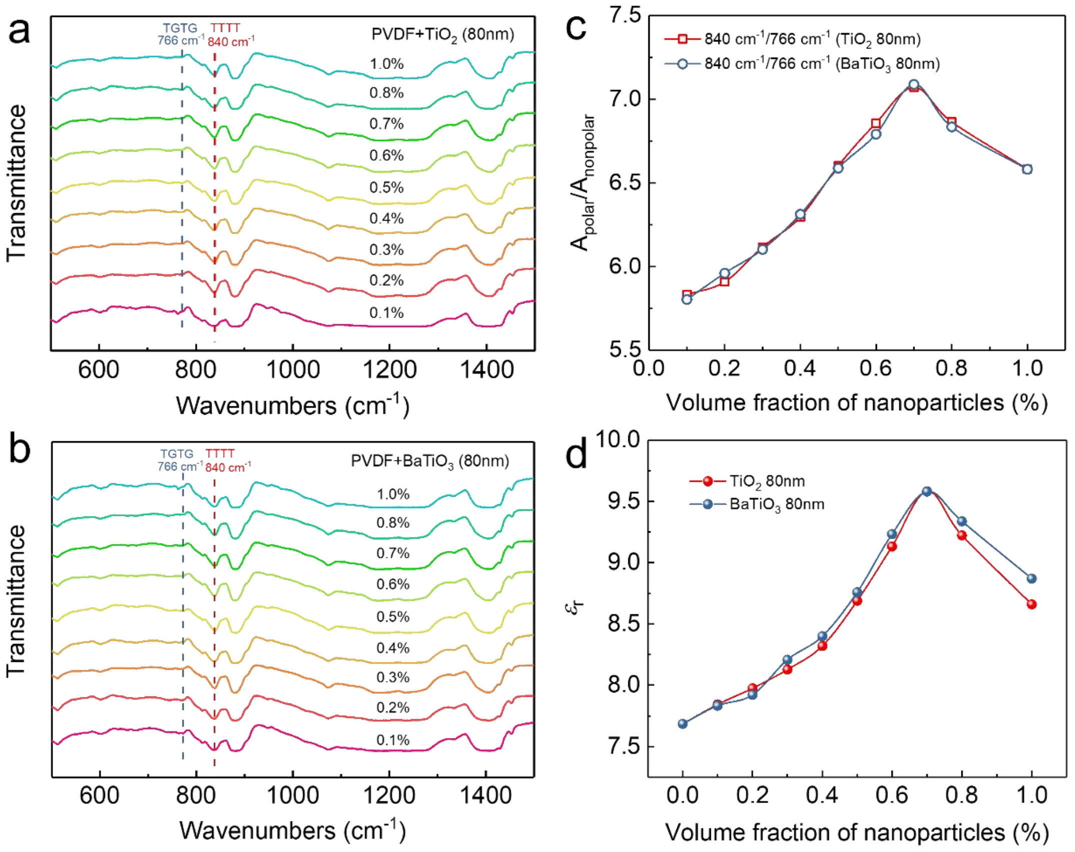

Supplementary Fig. 12 | a, b, FTIR spectra of the (a) PVDF-TiO_2 and (b) PVDF-$BaTiO_3$ nanocomposites with different volume fractions of nanoparticles. c, The calculated ratio of absorbance intensities (A_{840} and A_{766}) at 840 cm^{-1} and 766 cm^{-1} in the nanocomposites with different volume fractions of nanoparticles. d, Variation of dielectric constant as a function of volume fraction of different nanoparticles for nanocomposites.

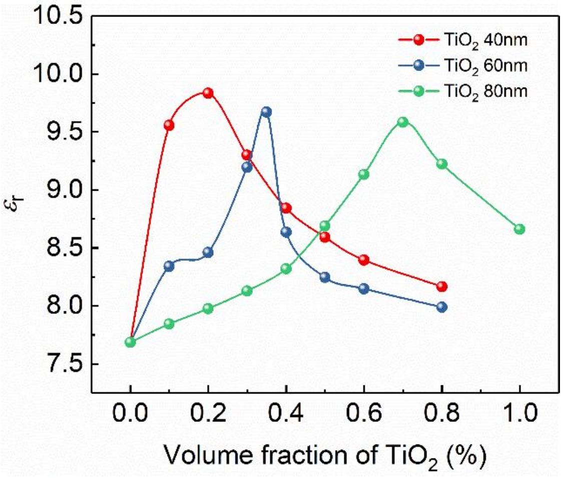

Supplementary Fig. 13 | Variation of dielectric constant as a function of volume fraction of TiO_2 nanoparticles with different diameters.

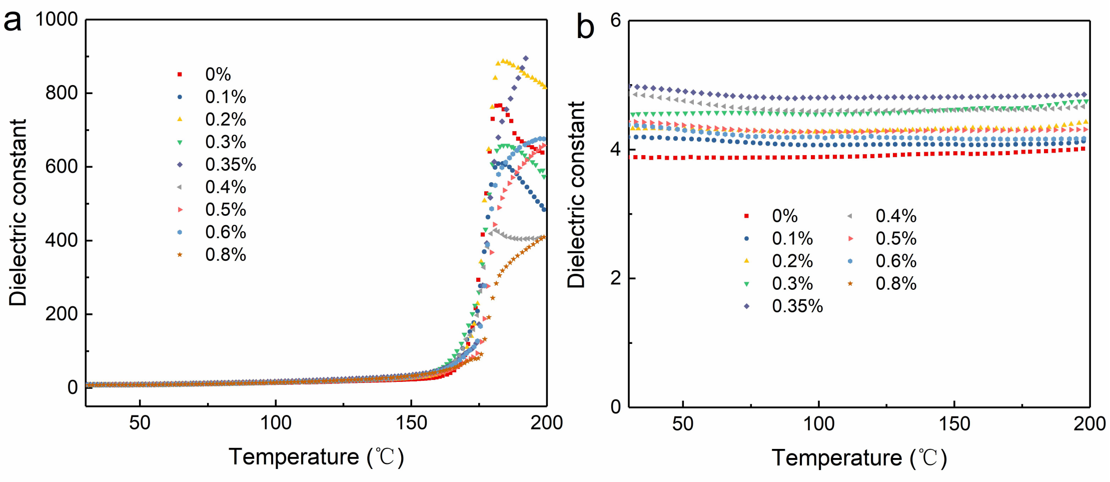

Supplementary Fig. 14 | The dielectric properties of nanocomposites under varying temperatures. a, The variation of dielectric constant with varying temperatures for PVDF-TiO_2 nanocomposites. **b**, The variation of dielectric constant with varying temperatures for PEI-TiO_2 nanocomposites.

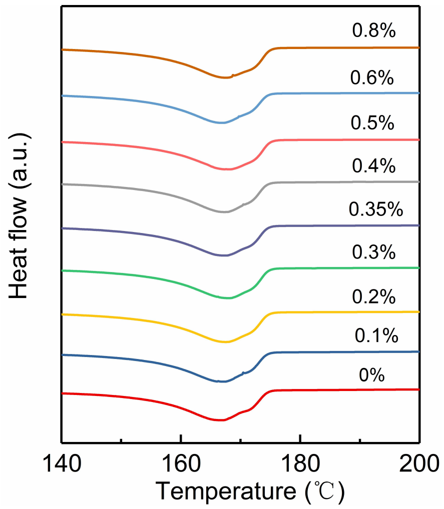

Supplementary Fig. 15 | The differential scanning calorimetry (DSC) curves of PVDF-TiO_2 nanocomposites with varying volume fractions of nanoparticles. The melting temperature of PVDF-TiO_2 nanocomposites determined from the DSC curves is around 170 ^{o}C.

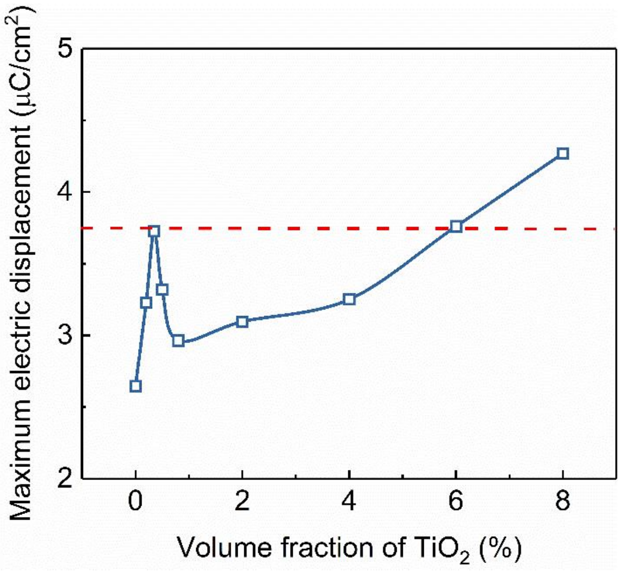

Supplementary Fig. 16 | Variation of electric displacement at electric field of 200kV/mm as a function of volume fraction of TiO_2 nanoparticles. A high loading of 6 vol.% TiO_2 nanoparticles, exceeding the concentration range of polar interfacial effect, also endows a similar enhancement of D by contributions from the intrinsic high ε_r of TiO_2 and interfacial space charge[9], however, the E_b value is notably decreased to 440 kV/mm, because the high loading intensifies the aggregations of nanoparticles and results in more structure imperfections.

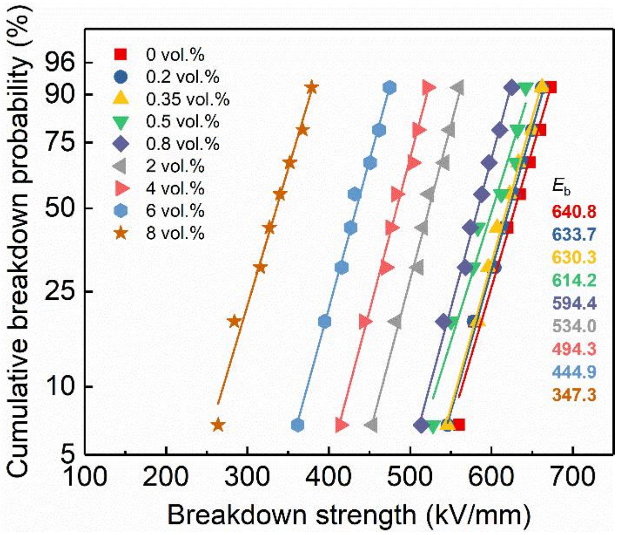

Supplementary Fig. 17 | Failure probabilities of breakdown strength deduced from Weibull distribution for PVDF-TiO$_2$ nanocomposites. The experimental breakdown strength of polymer nanocomposites is analyzed by the Weibull equation as $P(E)=1\text{-}\exp[-(E/E_b)^{\beta}]$, where $P(E)$ is the cumulative probability of electric failure, E is experimental breakdown strength, E_b is a scale parameter refers to the breakdown strength at the cumulative failure probability of 63.2%, which is also regarded as the characteristic breakdown strength, and β is the Weibull modulus associated with the linear regressive fit of the distribution.[10]

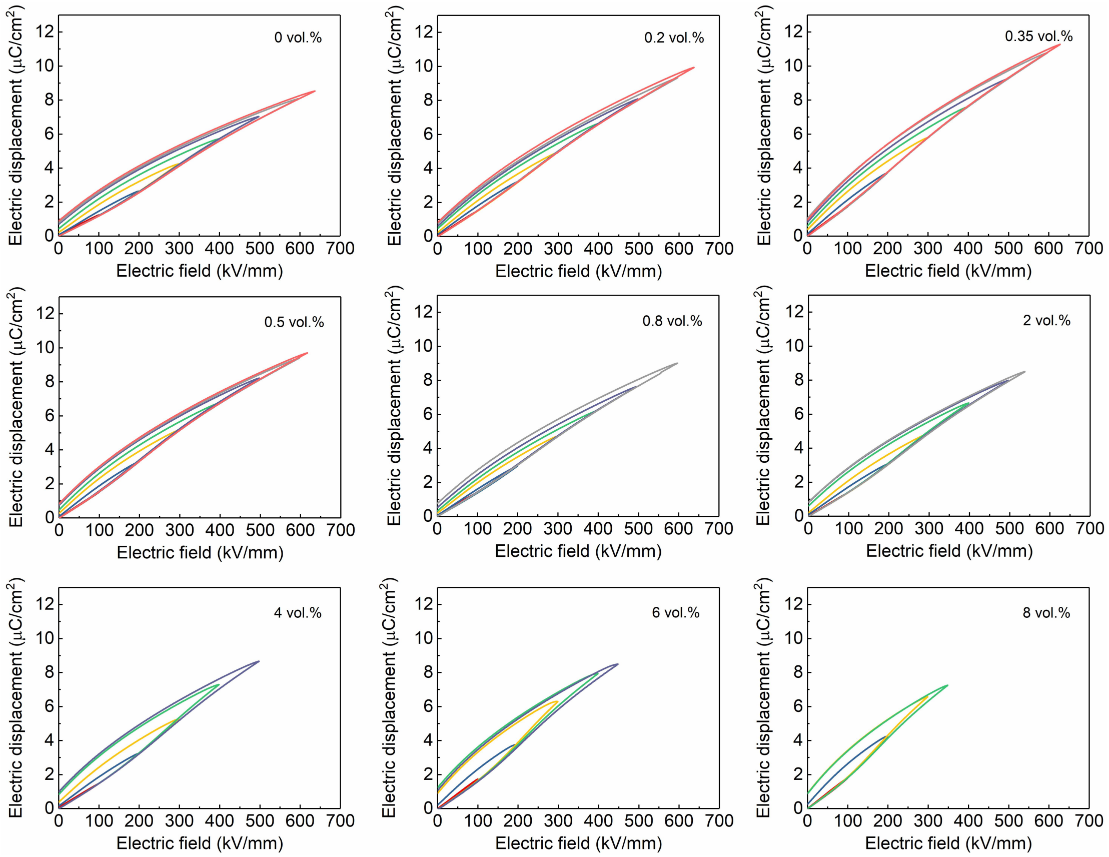

Supplementary Fig. 18 | Electrical displacement (*D*) - electric field (*E*) loops for PVDF-TiO_2 nanocomposites with different volume fractions of TiO_2 nanoparticles. Based on the *D*-*E* loop, the energy density (U_d) is calculated by $U_d = \int_{D_{\max}}^{D_r} E\, dD$, where D_r and D_{max} are the remnant electrical displacement and maximum electrical displacement, respectively.

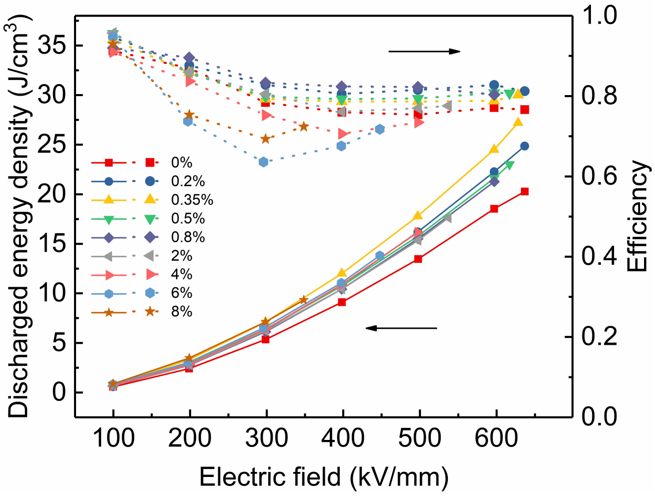

Supplementary Fig. 19 | Variation of discharged energy density and efficiency with the electric field for PVDF-TiO_2 nanocomposites with different volume fractions of TiO_2 nanoparticles.

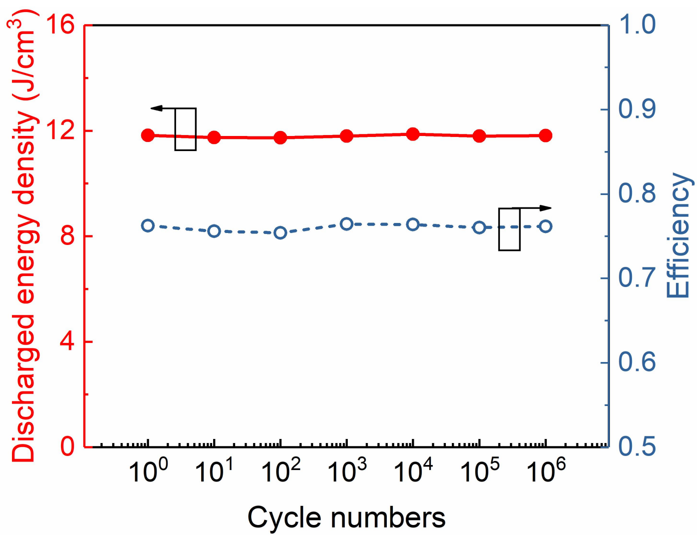

Supplementary Fig. 20 | Cyclic stability of energy density and energy efficiency of PVDF-TiO_2 nanocomposites with with 0.35 vol.% of TiO_2 nanoparticles under electric field of 400 kV/mm.

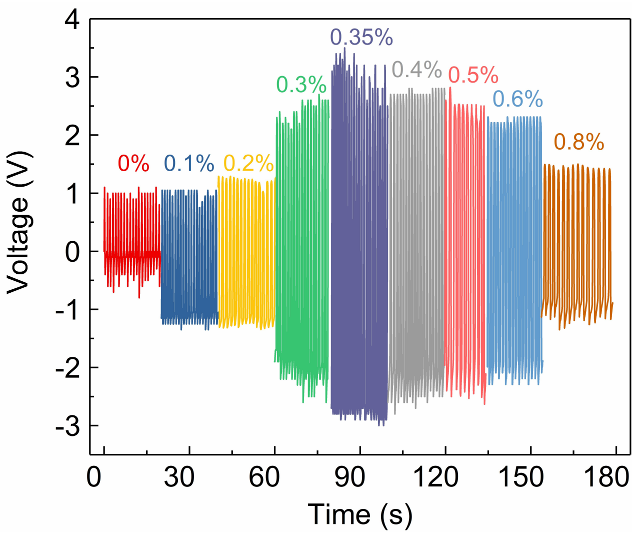

Supplementary Fig. 21 | Open-circuit voltage output signals of the PVDF-TiO_2 nanocomposites with varying volume fractions of nanoparticles under a pressing force of 10N.

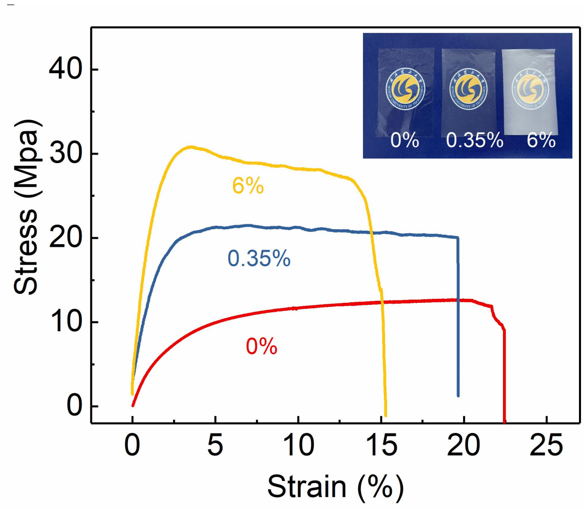

Supplementary Fig. 22 | The stretchability and transparency of polymer nanocomposites. The strain-stress curves of pristine PVDF, dilute nanocomposite with 0.35 vol.% of TiO_2 nanoparticles and high loading nanocomposite with 6 vol.% of TiO_2 nanoparticles, the inset shows the photo images of the three samples.

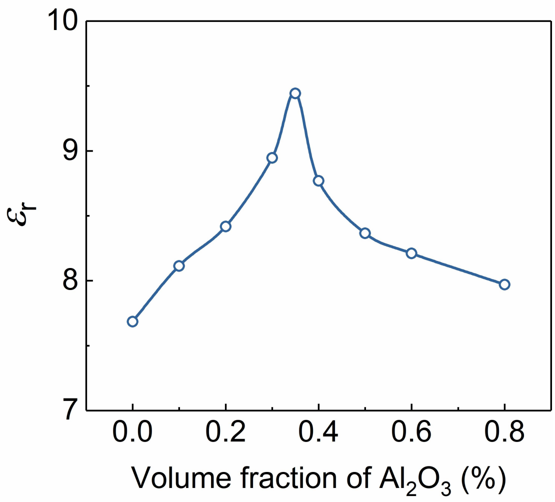

Supplementary Fig. 23 | Variation of dielectric constant as a function of volume fraction of Al_2O_3 nanoparticles (in diameter of 60nm) for PVDF-Al_2O_3 nanocomposites.

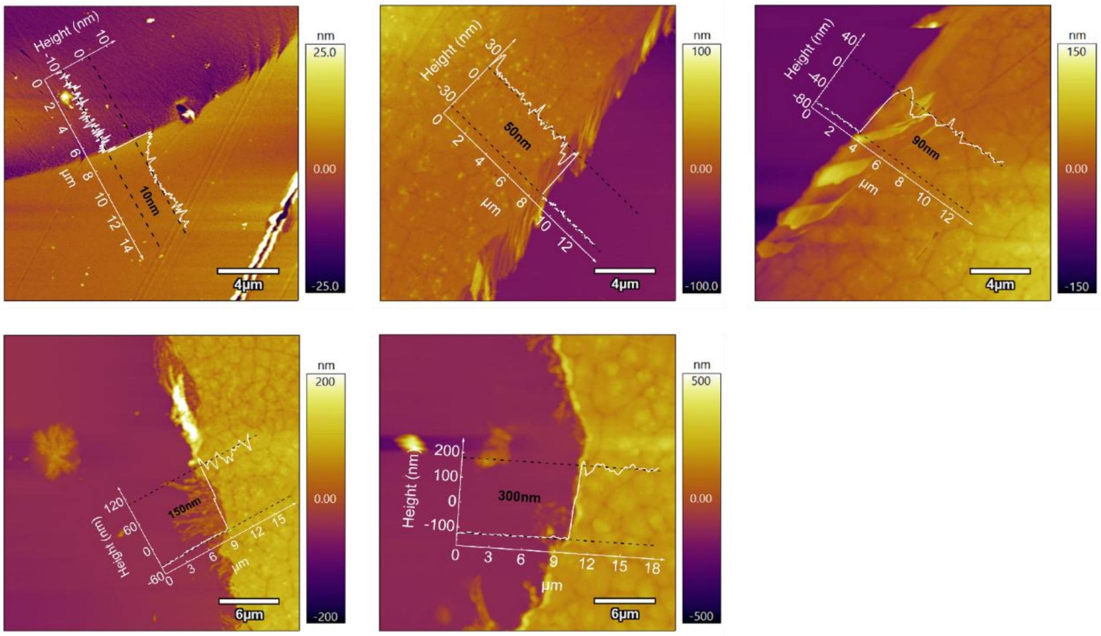

Supplementary Fig. 24 | AFM topography image of the PVDF thin film on the Al_2O_3 layer with different film thicknesses.

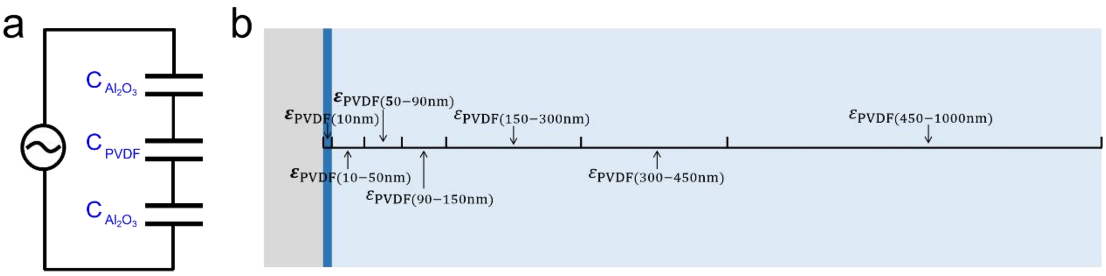

Supplementary Fig. 25 | Calculation of dielectric constant of interfacial PVDF layers with varying distances from the Al_2O_3 surface. a, Schematic illustration of the series connection of Al_2O_3 films and PVDF film. **b**, Schematic illustration of the PVDF film layers with different distances (x nm) from the Al_2O_3 surface and corresponding dielectric constant ($\varepsilon_{\mathrm{PVDF(x\,nm)}}$), $\varepsilon_{\mathrm{AlO}}$ indicates the dielectric constant of Al_2O_3. Based on the measured overall dielectric constant of layered Al_2O_3/PVDF/Al_2O_3 nanocomposite ($\varepsilon_{\mathrm{AlO/PVDF}(t\,\mathrm{nm})\mathrm{/AlO}}$) with varying PVDF film thickness (t nm) (Fig 5b), the PVDF film with different distances from the Al_2O_3 surface can be determined by the series law of capacitor. Take the Al_2O_3(10nm)/PVDF(t nm)/Al_2O_3(10nm) nanocomposite as an example, the dielectric constant can be expressed as

$$\frac{30}{\varepsilon_{\mathrm{AlO(10nm)/PVDF(10nm)/AlO(10nm)}}}=\frac{10}{\varepsilon_{\mathrm{AlO}}}+\frac{10}{\varepsilon_{\mathrm{PVDF(10nm)}}}+\frac{10}{\varepsilon_{\mathrm{AlO}}},$$

$$\frac{70}{\varepsilon_{\mathrm{AlO(10nm)/PVDF(50nm)/AlO(10nm)}}}=\frac{10}{\varepsilon_{\mathrm{AlO}}}+\frac{10}{\varepsilon_{\mathrm{PVDF(10nm)}}}+\frac{40}{\varepsilon_{\mathrm{PVDF(10-50nm)}}}+\frac{10}{\varepsilon_{\mathrm{AlO}}},$$

$$\frac{110}{\varepsilon_{\mathrm{AlO(10nm)/PVDF(90nm)/AlO(10nm)}}}=\frac{10}{\varepsilon_{\mathrm{AlO}}}+\frac{10}{\varepsilon_{\mathrm{PVDF(10nm)}}}+\frac{40}{\varepsilon_{\mathrm{PVDF(10-50nm)}}}+\frac{40}{\varepsilon_{\mathrm{PVDF(50-90nm)}}}+\frac{10}{\varepsilon_{\mathrm{AlO}}},$$

…… etc.

Thus, the distance-dependent dielectric constant of interfacial PVDF layers were calculated as shown in Fig 5c.

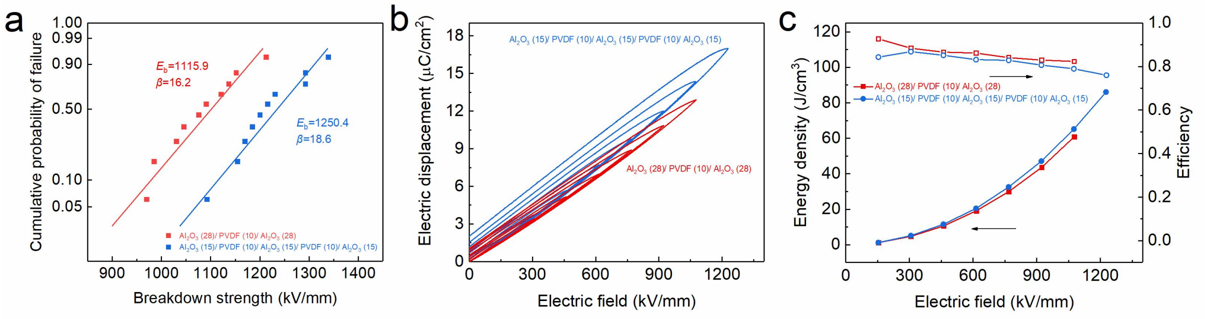

Supplementary Fig. 26 | Breakdown and energy storage properties of nanolaminated nanocomposites containing one and two PVDF bound polar layers. a, The Failure probabilities of breakdown strength deduced from Weibull distribution for Al_2O_3 (28 nm)/PVDF (10 nm)/Al_2O_3 (28 nm) and Al_2O_3 (15 nm)/PVDF (10 nm)/Al_2O_3 (15 nm)/PVDF (10 nm)/Al_2O_3 (15 nm) nanolaminated nanocomposites. **b**, *D*-*E* loops for Al_2O_3 (28 nm)/PVDF (10 nm)/Al_2O_3 (28 nm) and Al_2O_3 (15 nm)/PVDF (10 nm)/Al_2O_3 (15 nm)/PVDF (10 nm)/Al_2O_3 (15 nm) nanolaminated nanocomposites under different electric fields. **c**, Variation of discharged energy density and energy efficiency with the electric field for Al_2O_3 (28 nm)/PVDF (10 nm)/Al_2O_3 (28 nm) and Al_2O_3 (15 nm)/PVDF (10 nm)/Al_2O_3 (15 nm)/PVDF (10 nm)/Al_2O_3 (15 nm) nanolaminated nanocomposites.

Supplementary references

1. Soergel, E. Piezoresponse force microscopy (PFM), *J. Phys. D: Appl. Phys.* **44**, 464003 (2011).
2. Cheng, S. et al. Unexpected molecular weight effect in polymer nanocomposites, *Phys. Rev. Lett.* **116**, 038302 (2016).
3. Zhou, Z. & Abe, A. A RIS treatment of the mean-square dipole moment of PMMA chains in consideration of the pendant ester group orientations. *Polymer* **45**, 1313-1320 (2004).
4. Tretinnikov, O. N. & Ohta, K. Conformation-sensitive infrared bands and conformational characteristics of stereoregular poly(methyl methacrylate)s by variable-temperature FTIR spectroscopy. *Macromolecules.* **35**, 7343-7353 (2002).
5. Tretinnikov, O. N. Conformational characteristics of stereoregular PMMA and of the stereocomplex: new insights from FTIR measurements. *Macromol. Symp.* **203**, 57-70 (2003).
6. Dang, Z. M. et al. Fundamentals, processes and applications of high-permittivity polymer-matrix composites. *Prog. Mater. Sci.* **57**, 660-723 (2012).
7. Bruggeman, D. A. G. The calculation of various physical constants of heterogeneous substances. I. The dielectric constants and conductivities of mixtures composed of isotropic substances. *Ann. Phys*. **416**, 636-791 (1935).
8. Zakri, T.; Laurent, J.-P.; Vauclin, M. Theoretical evidence for Lichtenecker's mixture formulae' based on the effective mediumtheory. *J. Phys. D: Appl. Phys.* **31**, 1589-1594 (1998).
9. Shen, Z. et al. Space charge effects on the dielectric response of polymer nanocomposites. *Appl. Phys. Lett.* **111**, 92901 (2017).
10. Jiang, Y. et al. Ultrahigh breakdown strength and improved energy density of polymer nanocomposites with gradient distribution of ceramic nanoparticles. *Adv. Funct. Mater.* **30**, 1906112 (2019).
